# Daily life mobility detects frailty, falls, and functioning in ADT-treated prostate cancer survivors

**DOI:** 10.21203/rs.3.rs-4402624/v1

**Published:** 2024-05-30

**Authors:** Kerri Winters, Deanne Tibbitts, Martina Mancini, Sydnee Stoyles, Nathan Dieckmann, Julie Graff, Mahmoud El-Gohary, Fay Horak

**Affiliations:** OHSU; OHSU; OHSU; OHSU; OHSU; Oregon Health & Science University; Clario; OHSU

## Abstract

**Background:**

Androgen deprivation therapy (ADT) increases the risk of frailty, falls, and, poor physical functioning in prostate cancer survivors. Detection of frailty is limited to self-report instruments and performance measures, so unbiased tools are needed. We investigated relationships between an unbiased measure – daily life mobility – and ADT history, frailty, falls, and functioning in ADT-treated prostate cancer survivors.

**Methods:**

ADT-treated prostate cancer survivors (N=99) were recruited from an exercise clinical trial, an academic medical center, and the community. Participants completed performance measures and surveys to assess frailty, fall history, and physical functioning, then wore instrumented socks to continuously monitor daily life mobility. We performed a principal component analysis on daily life mobility metrics and used regression analyses to investigate relationships between domains of daily life mobility and frailty, fall history, and physical functioning.

**Results:**

Daily life mobility metrics clustered into four domains: Gait Pace, Rhythm, Activity, and Balance. Worse scores on Rhythm and Activity were associated with increased odds of frailty (OR 1.59, 95% CI: 1.04, 2.49 and OR 1.81, 95% CI: 1.19, 2.83, respectively). A worse score on Rhythm was associated with increased odds of ≥1 falls in the previous year (OR 1.60, 95% CI: 1.05, 2.47). Worse scores on Gait Pace, Rhythm, and Activity were associated with worse physical functioning. Mobility metrics were similar between current and past users of ADT.

**Conclusions:**

Continuous passive monitoring of daily life mobility may identify prostate cancer survivors who have or are developing risk for frailty, falls, and declines in physical functioning.

## Introduction

Nearly half of all prostate cancer survivors will be treated with androgen deprivation therapy (ADT),^1, 2^ a mainstay treatment that can slow cancer progression and increase survival.^3–6^ However, ADT has substantial side effects, including loss of muscle mass,^7, 8^ fatigue,^4, 5, 9–11^ impaired cognitive^9, 12, 13^ and physical functioning (e.g., slowed gait, instability, weakness),^14–16^ and increased risk of falls and frailty.^3, 5, 17, 18^

Frailty is an overall weakened physiological state usually associated with advanced age, which can be measured as a phenotype consisting of 5 criteria (exhaustion, weakness, slowness, sarcopenia and inactivity). It is linked to increased risk of hospitalization, disability, falls, and early death.^19–27^ Frailty is commonly measured using geriatric assessment, performance measures, and/or questionnaires,^28^ which may be time-consuming or lack sensitivity to identify patients early enough to intervene. Performance measures, such as walking speed, are typically conducted in a laboratory or clinical setting, where patients may be motivated to perform at their best. Questionnaires like the FRAIL scale^29^ are also prone to bias and underreporting, as a person will screen positive for frailty only when they perceive that they have limited functioning. The development of unbiased measures of actual functioning, such as how a person moves about in their daily life, would improve upon existing frailty assessments and potentially improve the ability to detect frailty associated with ADT.

Wearable technologies enable continuous, unobtrusive monitoring of mobility while people go about their daily activities. Compared to questionnaires or performance measures, assessing daily life mobility may better detect decrements in activity, gait, and balance that could lead to frailty. Studies suggest that mobility metrics captured with wearable sensors may discriminate between healthy individuals and those with neurologic diseases better than laboratory measures of gait and balance,^30, 31^ and may be able to predict future falls.^32^ We hypothesized that continuous monitoring of daily life mobility could detect physical declines related to ADT; however, this approach has not been tested in prostate cancer survivors.

The goals of this study were to 1) characterize relationships between ADT history and selected daily life mobility metrics; 2) identify domains of daily life mobility; and 3) examine relationships between domains of daily life mobility and frailty, falls, and physical functioning in prostate cancer survivors.

## Methods

### Study design and sample

We conducted an ancillary study to a fall prevention exercise trial^33^ in ADT-treated prostate cancer survivors, to relate daily life mobility metrics at baseline to ADT history, frailty, fall history, and physical functioning. We recruited participants from an exercise trial (as previously described^33^), emailed study information to people in a research repository, mailed or emailed patients identified from the hospital tumor registry at our academic medical center, and engaged prostate cancer support groups.

People were eligible if they had prostate cancer of any disease stage, received at least 6 months of ADT within the last 10 years, and had no concurrent treatment or conditions that contraindicated participation in performance testing. Participants from the exercise trial met additional criteria of having a history of falls in the last year or having fall risk (i.e., 3-meter timed up and go (3m TUG) time ≥ 12.0 seconds or 5-time chair stand time ≥ 10.0 seconds)^33^. We categorized participants as current (i.e., ADT in the past year) or past (i.e., > 1 year since ADT) users of ADT. This study was performed in accordance with the ethical standards of the Helsinki Declaration and conducted after approval by the Oregon Health & Science University institutional review board (IRB#18354).

### Procedures

Participants were screened for eligibility, provided written informed consent, and then attended an in-person study visit. Participants completed a series of performance measures, including a 4-meter walk, 5-time chair stand, and 3m TUG. At the study visit, participants received socks instrumented with inertial sensors (APDM Wearable Technologies, a Clario company, Portland, OR); description of the instrumented socks has been published.^30^ Instrumented socks were worn continuously during waking hours for 3–7 days to passively monitor daily life mobility. After completing the surveillance period, participants rated ease of use by responding to the following question: “I found the socks easy to use” on a 1–7 Likert scale, where 1 = strongly disagree and 7 = strongly agree. After the study visit, questionnaires were completed electronically at home. Self-report data included demographic characteristics, cancer and treatment history, comorbidities (Charlson Comorbidity Index^34, 35^), falls in the last year, perceived frailty (FRAIL scale^29^), perceived physical functioning (EORTC-QLQ-C30^36^), fatigue (SF-36 vitality subscale^37, 38^), and physical activity (CHAMPS^39^). Disease severity (metastatic versus non-metastatic) was abstracted from participant medical records.

### Outcomes

#### Daily life mobility metrics.

Daily life mobility was collected using instrumented socks worn on the feet.^30^ Daily life mobility metrics were calculated from the inertial sensors using previously validated proprietary algorithms from APDM,^40–42^ followed by calculation of metrics within each gait bout. Gait bouts were at least 3 seconds long and contained at least 3 steps.^43^ Summary metrics were derived by averaging metrics across all gait bouts from at least 3 days of wear.

We chose 12 daily life mobility metrics based on prior work^30, 43^ (Suppl. Table 1) that represented different aspects of gait quantity and quality for initial comparisons of participants based on ADT history and as inputs for the principal component analysis.

#### Frailty.

Objectively measured frailty was assessed using the Fried frailty phenotype; measures and cutoffs for each frailty criterion have been published.^33^ Perceived frailty was assessed using the FRAIL scale.^29^ Frailty categories were defined as: ≥3 criteria = frail; 1–2 criteria = pre-frail; 0 criteria = robust.

#### Falls.

A fall was defined as unintentionally coming to rest on the ground or some other lower level, not as a result of a major intrinsic event (e.g., stroke or syncope) or overwhelming hazard, a standard definition.^44^ Those who reported a fall in the past year also reported the number of falls.

#### Physical functioning.

Perceived physical functioning was assessed using the physical functioning subscale of the EORTC-QLQ-C30.^36^ Objectively measured physical functioning was assessed using 3m TUG, a reliable^45^ and widely accepted clinical measure of mobility that times how long it takes a person to rise from a chair, walk 3m, turn around, return, and sit in the chair.^46^

### Statistical analysis

Sample characteristics were summarized using descriptive statistics, while current versus past ADT users were compared using t-tests, Chi-squared tests of proportion, and Fisher’s Exact Test. Daily life mobility metrics were compared between current and past ADT users using t-tests. To identify domains of daily life mobility, we performed a principal component analysis (PCA) on the 12 daily life mobility metrics using the psych package in R.^47^ PCA components with eigenvalues greater than 1.0 were extracted, and we compared orthogonal and oblique component rotations to maximize interpretability. Individual component scores for each participant were calculated from the final PCA model to use as independent variables in multiple regression models to describe the relationship between PCA components (“daily life mobility domains”) and outcomes of interest.

To examine relationships between daily life mobility and frailty, falls, and physical functioning, we fit multivariable models, adjusting for age and disease severity. Objective frailty and fall history were tested for and modeled using proportional odds logistic regression, which is a special case of ordinal logistic regression allowing assessment of differences across all categories of frailty or faller status in a single model. Through the assumption of proportionality (i.e., odds ratio is constant across all comparisons; verified by the Brant test^48^), the odds ratio predicts increased frailty (both robust versus pre-frail/frail and robust/pre-frail vs frail) as PCA scores change, and increased fall risk (both any falls (≥1 fall) vs no falls and recurrent falls (≥2 falls) vs one or no falls). Physical functioning outcomes were modeled using linear regression. Analyses were performed using R v.4.2.2^49^ and alpha was set at 0.05.

## Results

### Sample characteristics

We enrolled 99 prostate cancer survivors (mean age: 73.0 +/− 7.3 years) (Table 1). Participants were primarily white (94%), college educated (75%), overweight (mean BMI: 28.2), had non-metastatic prostate cancer (73%), and were an average of 5.6 years past diagnosis. More participants were current users of ADT than past users (65% versus 35%). A majority of participants met objective criteria for being frail or pre-frail (75%), however, only 36% of participants met criteria based on self-report. Primary contributors to objective frailty classification were weakness (n = 57) and slowness (n = 38). Thirty-five percent of participants had fallen at least once in the last year, with 13% of participants reporting 2 or more falls. A higher proportion of current ADT users had metastatic disease than past users (41% vs 3%, respectively). Current ADT users also had significantly more comorbidities but did not differ on any other characteristics, including age.

#### ADT history and daily life mobility metrics

Participants wore instrumented socks for a mean of 6.5 days, exceeding the minimum wear time of 3 days needed to characterize daily life mobility. Participants rated the socks as easy to use (mean rating = 6.2 on a scale of 1–7; n = 78). Before performing the principal component analysis (PCA), we compared the 12 daily life mobility metrics between current and past ADT users. Of the 12 metrics, only pitch of the foot at initial contact was significantly different between groups (Suppl. Table 2); therefore, we performed the PCA using a pooled sample of current and past ADT users.

### Domains of daily life mobility and principal component analysis

**Figure 1:**
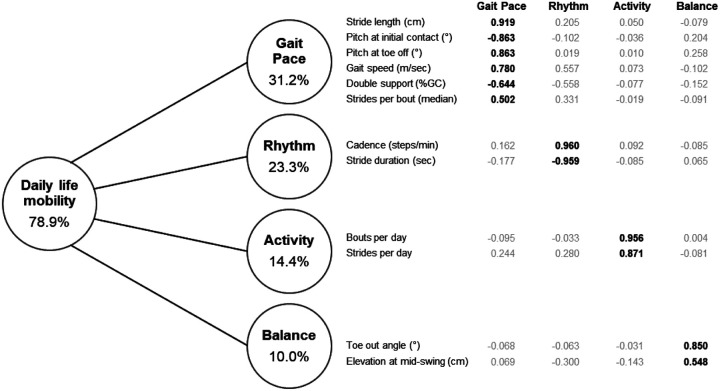
Model of daily life mobility for prostate cancer survivors treated with androgen deprivation therapy. A principal component analysis of 12 mobility metrics with varimax rotation produced 4 orthogonal domains of mobility. Factor loadings are listed in order of importance, and relevant factor loadings are bolded. Values inside the circles indicate the proportion of total variance explained by each domain. %GC, percent of gait cycle.

We next conducted PCA on the 12 daily life mobility metrics, which yielded four orthogonal components (“daily life mobility domains”) that accounted for 78.9% of the variance in mobility metrics (Fig. 1). The domains were classified as “Gait Pace” (31.2% of total variance), “Rhythm” (23.3%), “Activity” (14.4%), and “Balance” (10.0%). The loading threshold was empirically set at 0.4 or higher, and only two cross-loading factors were observed (gait speed and double support; Fig. 1).

The “Gait Pace” domain is comprised of high loadings on six metrics that describe or contribute to gait speed: a lower score on Gait Pace corresponds to shorter stride length, shallower angles of heel strike and toe-off, slower gait speed, larger proportion of the gait cycle spent in double support, and fewer strides per gait bout. The “Rhythm” domain is comprised of high loadings on two metrics that contribute to the timing of the gait cycle: a lower score on Rhythm corresponds to slower cadence (fewer steps per minute), and longer stride duration (more time needed to take a single stride). The “Activity” domain is comprised of high loadings on two metrics that describe the average amount of gait: a lower score on Activity corresponds to fewer gait bouts per day and fewer strides per day. The “Balance” domain is comprised of two variables that, when elevated, are characteristic of a gait pattern with a wider stance, which typically reflects an adaptation to better stabilize the body while walking and thus may indirectly indicate poorer balance.^50, 51^ A higher score on Balance corresponds to a larger toe-out angle (related to greater stride width) and higher elevation of the feet at mid-swing.

### Associations of daily life mobility domains with frailty, falls, and physical functioning

**Figure 2:**
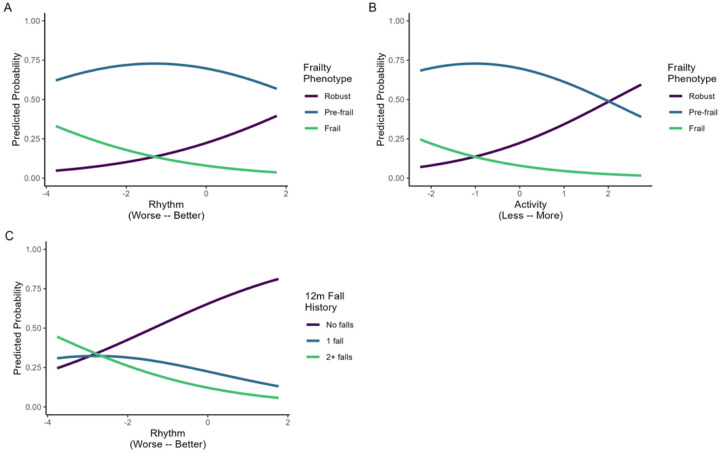
Predicted probabilities of frailty and fall history from domains for daily life mobility in prostate cancer survivors treated with androgen deprivation therapy. Domains of daily life mobility are plotted against the predicted probability of membership in one of three categories of frailty (A, B) or fall history (C). Panel (A): The predicted probability of frailty and pre-frailty decline as Rhythm improves. Panel (B): The predicted probability of frailty and pre-frailty decline as Activity increases. Panel (C): The predicted probability of having no falls in the previous 12 months increases as Rhythm improves. 12m, 12 months.

Worse scores on domains of Rhythm and Activity were significantly associated with increased odds of objectively measured frailty (Table 2). Every 1-point decline in the Rhythm and Activity domains resulted in 1.59 times (95% CI: 1.04, 2.49) and 1.81 times (95% CI: 1.19, 2.83) increased odds, respectively, of being classified as frail or pre-frail compared to robust, with the same proportional increase in risk of being classified as frail compared to pre-frail or robust. Figures 2A and 2B show the predicted probabilities of membership in each of the three frailty categories across the domain ranges for Rhythm and Activity. In Fig. 2A, worse performance in Rhythm is associated with the highest likelihood of being frail. In Fig. 2B, lower Activity is associated with the highest likelihood of being frail. Gait Pace and Balance had inconclusive odds ratios. Odds ratios were unchanged after adjusting for age and metastatic disease.

The domain of Rhythm was uniquely associated with falls in the previous year (Table 2). Every 1-point decline in the Rhythm domain resulted in 1.60 times increased odds of having one or more falls in the past year compared to not falling (95% CI: 1.05, 2.47) with the same proportional increase in risk for being a recurrent faller compared to never or one-time fallers (1.60; 95% CI: 1.05, 2.47). In Fig. 2C, worse Rhythm is associated with the highest likelihood of being a recurrent faller. Gait Pace, Activity, and Balance had inconclusive odds ratios. Odds ratios were unchanged after adjusting for age and metastatic disease.

Lower scores in Gait Pace, Rhythm, and Activity domains were associated with worse physical function. Gait Pace and Activity showed the strongest association with perceived physical function, with a 3-point decline for every 1-point worsening in Gait Pace or Activity, after controlling for age and metastatic disease. Lower scores in Gait Pace and Rhythm were associated with slower 3m TUG times, with a 1-point decrease in the Gait Pace domain corresponding to a 1.12 second (95% CI: 0.71, 1.54) slower 3m TUG time and a 1-point decrease in the Rhythm domain corresponding to a 0.78 second (95% CI: 0.36, 1.20) slower 3m TUG time (Table 3); associations were unchanged after adjusting for age and metastatic disease.

## Discussion

Using a novel wearable device to continuously and passively monitor daily life mobility, we found that several domains of mobility were significantly associated with frailty, falls, and physical functioning in prostate cancer survivors treated with ADT. Mobility metrics were similar among participants who were currently on ADT and participants who had been off ADT for at least one year. Daily life mobility metrics clustered into four domains of Gait Pace, Rhythm, Activity, and Balance. These domains were significantly associated with clinically important outcomes, suggesting that passively monitoring daily life mobility could provide a useful, objective marker to identify prostate cancer survivors who have or are developing risk for frailty, falls, and dependence.

Ours is the first study to measure daily life mobility in persons with cancer, a construct that may be a unique reflection of the impact of cancer and treatment on everyday functioning and falls risk. The domains of daily life mobility identified by PCA are consistent with the known side effects of ADT, further validating the utility of daily life mobility measurement to passively monitor for developing risk of frailty, falls and functional decline. Gait Pace and Rhythm accounted for the majority of variance in daily life mobility. The metrics within these domains, including gait speed, heel-strike angle, and toe-off angle, have been linked to fatigue and muscle weakness in populations with neurological diseases,^30, 31, 52^ but this is the first evidence that these mobility characteristics associate with worse clinical outcomes in prostate cancer survivors on ADT. Fatigue and deconditioning also contribute to low self-report activity levels in patients on ADT, and we observed fewer daily bouts and shorter bout length in participants in our sample who reported more falls, were frailer, and had lower functioning than participants with higher Activity scores.

ADT is a mainstay therapy for the treatment of prostate cancer, and while it markedly improves survival, many patients experience adverse effects that lead to frailty, falls, and functional decline.^3, 5, 14–18^ However, routine monitoring for signs of frailty or functional decline in clinical practice is lacking, which leaves survivors vulnerable to progressive declines and without opportunities for timely intervention. While assessment tools like the TUG test exist, administration in a clinical setting may be burdensome and difficult to implement often enough to detect early declines. While questionnaires like the FRAIL scale could fill this gap, our results show that survivors vastly underestimate their own frailty when compared to objective measures of frailty. The discrepancy between self-reported and objectively measured frailty underscores the need for objective, low-burden tools to detect decrements in gait quality and activity that could detect the onset of frailty. Indeed, the high acceptability and compliance to wearing the instrumented socks in our sample suggests that further investigation is warranted about the utility of instrumented socks as a clinical assessment tool. Wearable devices that measure daily life mobility, like instrumented socks, could potentially fill a gap in clinical practice by identifying patients at risk for frailty, falls, and dependence, which could provide data for shared decision-making between providers and patients around ADT management.^53^

Our study had several strengths. The use of a novel device to measure prostate cancer survivors’ gait quality and quantity at home provided us with an unbiased assessment of gait patterns during daily activities. We also captured an average of 6.5 days of daily mobility data, which provided us with a broad observation window for capturing natural variations in activities throughout the week and increased the likelihood that data were representative of each participant’s lifestyle. Our study also had limitations. Most participants had first enrolled in an exercise clinical trial, which was less likely to include men with limited functioning. As a cross-sectional study, we cannot ascertain whether daily life mobility is a cause or consequence of falls, frailty, and/or limited functioning. However, according to most conceptual models of aging, objective measures of strength, gait, and balance are the first signs that frailty, falls, and dependence may be developing.^54^ Also, racial diversity was limited, so our results may not be generalizable to all men treated with ADT.

In summary, our findings provide evidence that continuous passive monitoring of daily life mobility can detect frailty, falls, and functioning in prostate cancer survivors treated with ADT, which has important implications for understanding and preventing the adverse effects of ADT. Future work should investigate whether wearable sensors, such as instrumented socks, can detect changes in daily mobility over time and thus provide an objective, unobtrusive, and unbiased tool to monitor for mobility changes once men start ADT. Monitoring for declines in daily life mobility after ADT initiation could also provide a way to assess who is most at risk for frailty so interventions, such as those we are currently testing^33^, can be efficiently applied in resource constrained settings.

## Figures and Tables

**Table 1:** Sample demographics and characteristics by ADT status.

	Whole Sample		Current ADT Use	Past ADT Use	
	(N = 99)		(n = 64)	(n = 35)	
Characteristic	M (SD) or %	Range	M (SD) or %	M (SD) or %	p-value^a^
Age (yrs)	73.0 (7.3)	51.2–87.5	72.1 (7.2)	74.6 (7.3)	0.102
Race					
White	94%		97%	89%	0.328
African American/Black	1%		0%	3%	
Asian	2%		2%	3%	
More than 1 race	2%		2%	3%	
Other^b^	1%		0%	3%	
Hispanic (White and Non-White)	2%		0%	6%	0.123
Bachelors or higher	75%		70%	83%	0.258
Metastatic disease	27%		41%	3%	**< 0.001**
Time since diagnosis (yrs)	5.6 (4.7)	0.9–25.8	5.5 (4.8)	5.8 (4.5)	0.744
BMI (kg/m^2)	28.2 (4.2)	20.2–39.9	28.5 (4.5)	27.7 (3.8)	0.340
Charlson Comorbidity Index	3.8 (2.4)	0.0–11.0	4.4 (2.5)	2.8 (1.8)	**0.001**
Objective frailty					
Robust	25%		25%	26%	1.000
Pre-frail	65%		64%	66%	
Frail	10%		11%	9%	
Perceived frailty					
Robust	64%		59%	71%	0.454
Pre-frail or frail	36%		41%	29%	
Fall history (last 12 months)					
1 fall	22%		17%	31%	0.274
2 + falls	13%		14%	11%	
3m TUG (sec)	11.8 (2.5)	6.5–21.5	11.9 (2.5)	11.7 (2.4)	0.690
QLQ-C30 physical function	91.0 (12.7)	40.0–100.0	90.2 (13.2)	92.4 (11.7)	0.406
SmartSocks days of wear	6.5 (2.3)	3.0–25.0	6.1 (1.3)	7.1 (3.3)	0.092

Abbreviations: BMI, body mass index; QLQ-C30, Quality of Life Questionnaire Core 30; TUG, timed up and go

a Continuous variables compared using two-sample t-tests; categorical measures compared using Chi-squared tests of proportion or Fisher’s Exact Tests.

b Category includes “human” (n = 1).

**Table 2:** Odds ratios of increased frailty and falls as PCA components worsen.

	Objective Frailty		Fall History	
	Robust vs Pre-frail vs Frail		Non-faller vs 1 Fall vs 2+Falls	
	Unadjusted OR	Adjusted OR	Unadjusted OR	Adjusted OR
Independent Variables	(95% CI)	(95% CI)	(95% CI)	(95% CI)
PCA1 (Gait Pace)	1.48*	1.53*	0.96	1.00
	(0.95, 2.34)	(0.94, 2.53)	(0.62, 1.46)	(0.63, 1.58)
PCA2 (Rhythm)	1.59**	1.59**	1.60**	1.62**
	(1.04, 2.49)	(1.04, 2.49)	(1.05, 2.47)	(1.06, 2.52)
PCA3 (Activity)	1.81***	1.81***	1.04	1.05
	(1.19, 2.83)	(1.18, 2.84)	(0.68, 1.61)	(0.69, 1.63)
PCA4 (Balance)	0.94	0.90	1.11	1.11
	(0.62, 1.42)	(0.59, 1.37)	(0.71, 1.67)	(0.71, 1.69)
Age		0.98		0.99
		(0.92, 1.05)		(0.93, 1.05)
Metastatic disease		1.45		0.81
		(0.54, 4.00)		(0.29, 2.12)

Notes:

Odds ratios (OR) for PCA values show the odds of being more frail or having more falls for every 1-point decrease in PCA1–3 component score and every 1-point increase in PCA4 component score.

ORs for age show the odds of being more frail or having more falls for every 1 year increase in age.

ORs for metastatic disease show the odds of being more frail or having more falls if metastatic disease is present versus no metastatic disease.

Both objective frailty and 3-category faller status models were assessed for proportional odds using the Brant test. No evidence to reject the proportional odds was found.

Age was mean centered before adding to the models.

* p < 0.1;

** p < 0.05;

*** p < 0.01

**Table 3:** Odds ratios of perceived and objective physical function by components of daily life mobility.

	QLQ-C30 Physical Function		3m TUG (seconds)	
	Unadjusted β	Adjusted β	Unadjusted β	Adjusted β
Independent Variables	(95% CI)	(95% CI)	(95% CI)	(95% CI)
PCA1 (Gait Pace)	−2.06	−3.02**	1.12***	1.10***
	(−4.52, 0.39)	(−5.70, −0.34)	(0.71, 1.54)	(0.63, 1.57)
PCA2 (Rhythm)	−0.54	−0.68	0.78***	0.78***
	(−3.18, 2.10)	(−3.27, 1.90)	(0.36, 1.20)	(0.36, 1.20)
PCA3 (Activity)	−2.85**	−2.88**	0.04	0.04
	(−5.28, −0.42)	(−5.25, −0.51)	(−0.37, 0.46)	(−0.38, 0.46)
PCA4 (Balance)	−2.40*	−1.90	−0.12	−0.10
	(−4.86, 0.05)	(−4.32, 0.53)	(−0.53, 0.30)	(−0.52, 0.33)
Age		0.42**		0.01
		(0.05, 0.78)		(−0.05, 0.08)
Metastatic disease		−2.69		−0.19
		(−8.29, 2.91)		(−1.17, 0.80)

**Notes**:

Both outcomes were modeled using linear regression. Coefficients show the change for every 1-point decrease in PCA1–3 component score and every 1-point increase in PCA4 component score.

Age was mean centered before adding to the models.

* p < 0.1;

** p < 0.05;

*** p < 0.01

## Data Availability

The data underlying this article cannot be shared publicly due to the privacy of individuals that participated in the study. Deidentified data are available from the corresponding author upon request through a data use agreement for specific, approved analyses.
